# Prevalence of Congenital Anomalies and Predictive Factors in Pregnant Women in Bahrain: A Retrospective Analysis

**DOI:** 10.3390/life15040650

**Published:** 2025-04-15

**Authors:** Asal Buhasan, Leen Al Zayer, Lana Al-Jabery, Asmahan Mohamed, Abdulraoof Almadhoob, Entesar Zaman, Rafiea Jeddy

**Affiliations:** 1 School of Medicine, Royal College of Surgeons in Ireland, Busaiteen 15503, Bahrain; asal.buhasan@gmail.com (A.B.); leenzayer@gmail.com (L.A.Z.); aljaberylana@gmail.com (L.A.-J.); asmahan.m13@gmail.com (A.M.); 2 Neonatal Intensive Care Unit, Department of Pediatrics, Salmaniya Medical Complex, Manama 329, Bahrain; armadhoob@hotmail.com; 3 Department of Obstetrics & Gynaecology, Salmaniya Medical Complex, Manama 329, Bahrain; ealzaman@yahoo.com; 4 Department of Obstetrics & Gynaecology, Royal College of Surgeons in Ireland, Busaiteen 15503, Bahrain

**Keywords:** congenital anomalies, prevalence, risk factors, Bahrain

## Abstract

**Background and Objectives**: In Bahrain, congenital anomalies (CAs) account for 8.1% of the total deaths in children under 5, indicating a need to understand the patterns, predictors, and prevalence to improve antenatal standards of care and reduce the burden of disease. This study aimed to determine the prevalence and characteristics of CAs in Bahrain and investigate their association with measured neonatal and maternal risk factors. **Method**: In this five-year retrospective study, data from 31,615 neonates were analyzed, of which 354 had confirmed CAs. Stillbirths, abortions, and CAs discovered later in life were excluded from the study. **Results**: The prevalence of CAs was determined to be 1.1% (incidence of 11.2 per 1000 births), with 40.1% of the CAs affecting multiple systems. A statistically significant association was found between CAs and nationality, method of conception, gender, method of delivery, diabetes mellitus status, hypertension, and gestational age at birth. However, the association between CAs and multiple gestations was deemed statistically insignificant. **Conclusions**: As the first study exploring the associations between CAs and potential risk factors and outcomes in Bahrain, it provided a foundation for further exploration of the topic and insight into factors healthcare providers would target during preconception and antenatal care.

## 1. Introduction

The World Health Organization (WHO) defines congenital anomalies (CAs) as functional or structural abnormalities that develop during intrauterine life. They are one of the leading contributing factors to the global burden of disease, impacting an estimated 6% of newborns worldwide [1]. Anomalies can occur in isolation or as combined presentations involving multiple organ systems. They are further categorized as either major, manifesting as significant medical and social impairments, or minor, which have reduced impairments in neonatal development [2]. CAs contribute significantly to increasing neonatal disability, morbidity, and mortality, with an incidence of 7.9 million cases worldwide in 2023 and approximately 240,000 neonatal and 170,000 cases in children under five years [3]. 

Most CAs occur sporadically; however, there are identified genetic mutations, environmental exposures, and teratogenic agents linked with their prevalence [1]. Maternal risk factors, such as pre-gestational diabetes mellitus, antenatal infections, smoking, alcohol consumption, and chronic illnesses, have also been linked to the development of CAs. These factors can disrupt fetal development, reduce placental function, and impair metabolic exchanges between mother and fetus [4]. Therefore, understanding the connection between them is crucial for understanding the pathogenesis of CAs and implementing culturally relevant preventative screening measures.

In Bahrain, research regarding the incidence of CAs and identifiable maternal risk factors is limited. The most recent study was conducted between 1980 and 1990 and determined an anomaly rate of 2.7% [5]. Our study aims to provide updated insights regarding the incidence of CAs in Bahrain and the current identifiable maternal risk factors.

## 2. Materials and Methods

### 2.1. Study Population

This five-year retrospective cohort study included all live births at Salmaniya Medical Complex (SMC) in Bahrain from January 2018 to December 2022. According to the Ministry of Health’s records, SMC represents 33% of the total births in Bahrain, with a total of 1067 beds in the hospital, 106 of which are specifically for the Obstetrics and Gynaecology Department. Stillbirths and abortions were not included in this study due to the inability to confirm the presence of a CA. 

### 2.2. Data Collection

Data collection occurred in two phases. The first phase involved collecting maternal and neonatal data for confirmed or suspected anomalies from the labor ward registry. The second phase involved confirming the diagnosis of an anomaly through the hospital’s electronic medical records. CAs discovered later in life were not included in this study. 

Mothers of multiple pregnancies were included as one entry, while the neonates born were considered separately. This led to a difference in the total number of participants for maternal factors compared to neonatal/perinatal factors. However, if the same mother gave birth in different years, it was considered as different entries since some maternal factors, such as parity and age, changed.

The CAs were classified into eleven different systems: multiple (>2 systems), musculoskeletal (MSK), cardiology, oral and maxillofacial, central nervous system (CNS), gastrointestinal (GI), genitourinary (GU), ear, nose and throat (ENT), skin, respiratory, and ophthalmology. For each of these cases, maternal and neonatal risk factors were recorded from the labor ward registry. Maternal factors included maternal age at conception (<20 vs. 20–35 vs. ≥35 years), nationality (Bahraini vs. Non-Bahraini), parity (primipara, multipara: 2–4 vs. grandpara: ≥5), method of conception (spontaneous vs. induced), method of delivery (normal vaginal delivery vs. assisted vaginal delivery vs. cesarean section), previous baby with anomaly (yes vs. no), hypertension (yes vs. no), and diabetes mellitus status (pre-gestational diabetes mellitus vs. gestational diabetes mellitus vs. none). Neonatal factors included gender (male vs. female), gestational age at birth (term: 37 to 42 weeks vs. preterm: <37 weeks), neonatal birth weight (extremely low birth weight: <1 kg vs. very low birth weight: >1–<1.5 kg vs. low: >1.5–<2.5 kg vs. normal 2.5–4 kg vs. macrosomia > 4 kg), multiple gestations (yes vs. no), and neonatal outcome (discharged vs. perinatal death: ≤first 7 days of life vs. in-hospital death: death > 7 days of life). Perinatal death and in-hospital death results included only the neonates who died before discharge. The status of the babies after discharge was not investigated. 

### 2.3. Statistical Analysis

The collected data were transferred onto an Excel sheet and later exported to IBM’s SPSS software (SPSS v26) for statistical analysis. Firstly, descriptive statistics and frequency analysis were conducted to determine the incidence of CAs in the different systems and the characteristics of different maternal and perinatal risk factors. Secondly, chi-square tests were used to analyze the association between maternal nationality, method of conception, method of delivery, diabetes mellitus status, presence of hypertension, baby’s gender, multiple gestations, gestational age at birth, and CAs. The associations were analyzed and considered statistically significant if the *p*-value was <0.05.

### 2.4. Ethics Statement

Ethical approval was obtained for this study from the Research Committee for Government Hospitals, Bahrain (approval serial no. 92030923). The need for participant consent was waived by the Research Committee due to the data being anonymized.

## 3. Results

Throughout the duration of this study, 31,615 neonates were born at SMC. Based on the labor ward registry data, 399 were identified with or suspected to have a CA. Further investigations confirmed CAs in 354 neonates, resulting in a prevalence of 1.1% and an incidence of 11.20 per 1000.

**Figure 1:**
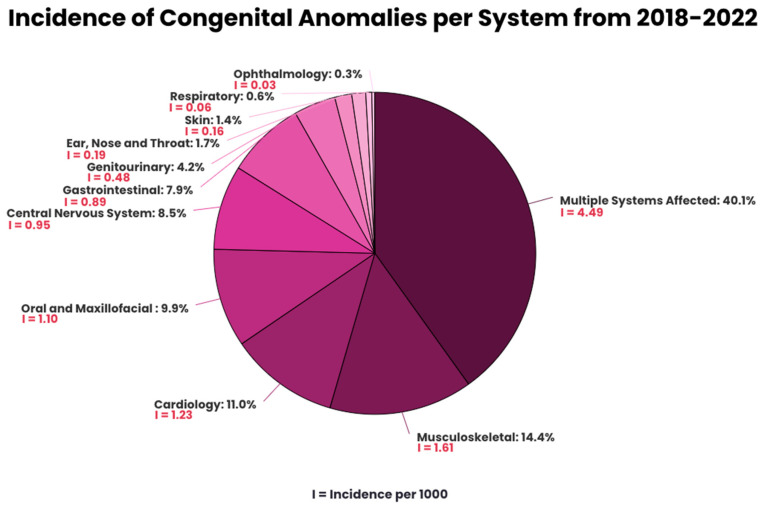
Incidence of congenital anomalies per system from 2018–2022.

CAs involving multiple systems were predominant, representing 40.1% of the anomalies, illustrated in Figure 1. For anomalies involving a single organ, MSK has the highest prevalence (14.4%), followed by cardiology (11.0%), oral and maxillofacial (9.9%), CNS (8.5%), GI (7.9%), GU (4.2%), ENT (1.7%), skin (1.4%), respiratory (0.6%), and ophthalmology (0.3%). The prevalence of specific types of anomalies is found in Appendix A (Table A1).

**Figure 2:**
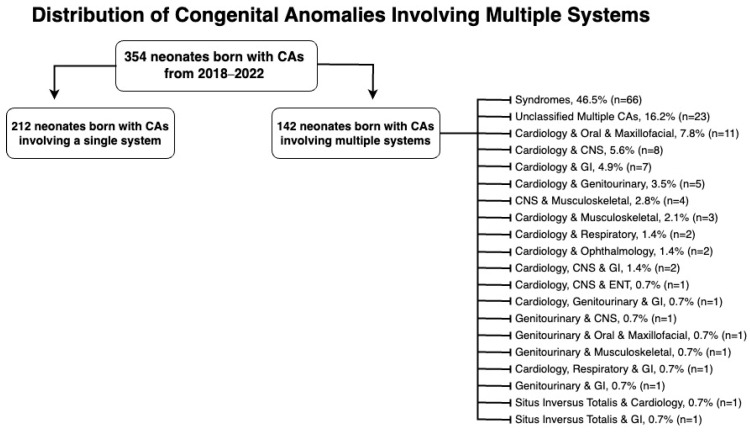
Distribution of congenital anomalies involving multiple systems.

Figure 2 categorizes multiple-system anomalies by the systems affected. Syndromes, with 66 cases, accounted for 46.5% of the multiple system congenital anomalies, while no definitive diagnosis was reached for 23 cases (16.2%), leaving them unclassified. Notably, the cardiovascular system was the most frequently affected in anomalies involving multiple systems, affecting 45 cases (31.7%) in total. 

Table 1 shows the association between maternal and perinatal factors with CAs. There was a statistically significant association between CAs and nationality, method of conception, method of delivery, gender, diabetes mellitus status, hypertension, and gestational age at birth. However, multiple gestations was deemed statistically insignificant with a *p*-value of 0.166. 

Table 2 portrays maternal characteristics in mothers whose babies had CAs. Regarding maternal age, 1.4% were <20 years old, 69.2% were 20–35 years old, and 29.3% were ≥35 years. Of note, 17 of the mothers with babies with Down syndrome were ≥35 years, while the remaining 5 were between 20–35. With regard to maternal parity, 119 mothers (33.9%) were primiparous, 210 (59.8%) were multiparous, and the least common was grandmultiparity with 22 mothers (6.3%). Of note, 8 women (2.3%) had a history of previous babies with CAs, while the remaining 346 (97.7%) did not.

Table 3 details the characteristics of the neonates with CAs. Among the neonates, 204 (57.3%) were males and 144 (40.7%) were females. Regarding gestational age, 65.0% were term deliveries, followed by preterm (27.7%), very preterm (6.2%), and extremely preterm (1.1%). The most common mode of delivery for the neonates with CAs was cesarean section, used in 206 deliveries (58.2%). This was followed by normal vaginal delivery in 137 cases (38.7%) and lastly, assisted vaginal delivery in 11 cases (3.1%). Neonatal birth weight distribution showed 221 babies (62.6%) with normal weight, 10 babies (2.8%) with high birth weight, 100 babies (28.3%) with low birth weight, 17 babies (4.8%) with very low birth weight, and 5 babies (1.4%) with extremely low birth weight. Among the neonates with CAs born during this study period, 16 (4.5%) were from multiple pregnancies, while the remaining 338 (95.5%) were from singleton pregnancies. After birth, 265 babies (74.9%) were discharged, 66 (18.6%) died perinatally, and 23 (6.5%) died in the in-hospital after the perinatal period. 

## 4. Discussion

### 4.1. Main Findings

The prevalence of CAs at SMC between 2018 and 2022 was determined to be 1.1% (incidence of 11.2 per 1000 births). Multiple-system CAs had the highest prevalence (21.5%), followed by syndromes and chromosomal abnormalities (18.6%), musculoskeletal (14.4%), and cardiovascular (11.0%). A statistically significant association was found between CAs and nationality, method of conception, method of delivery, gender, diabetes mellitus status, hypertension, and gestational age at birth. However, the association between CAs and multiple gestations was deemed statistically insignificant. 

### 4.2. Interpretation 

Our results show a similar prevalence rate to that of neighboring countries. While direct comparisons are limited due to a lack of available updated data, the most recent reported rates were Saudi Arabia (3.1%), Qatar (1.3%), Kuwait (1.3%), and UAE (2.1%) [6,7,8,9].

This study identified a distinct pattern in CA presentation with anomalies involving multiple systems having the highest prevalence (4.49 per 1000 births). This finding deviates from the general literature, which reports that approximately 75% of the CAs occur in isolation [10]. The WHO suggests that presentations involving multiple anomalies, specifically outside of syndromic associations, are considered uncommon, representing roughly 25% of the reported cases, similar to our study, which reported 21.5%. As our study only takes into account anomalies found at birth, this can lead to an underestimation in the number of chromosomal and syndromic anomalies.

The analysis of individual affected systems portrayed MSK anomalies as the most frequent (1.61 per 1000 births), followed by cardiology (1.23 per 1000 births). This finding conflicts with the previous literature, which reports cardiology, specifically congenital heart diseases (CHDs), as the most prevalent (8–9.5 per 1000 live births) [11]. Furthermore, the most recent study in Bahrain conducted by Al Arrayed in 1990 identified MSK, GU, and CHDs as the anomalies with the highest average incidences, reported as 2.07, 1.93, and 1.32 per 1000 births, respectively. In comparison to Al Arrayed’s paper, our study noted a decrease in CAs in all the systems with a particular reduction in GU. This is justified by undescended testes being the most common GU anomaly in Al Arrayed’s paper and common cardiac CAs presenting later in life, both not reported in this study [5,12].

Syndromes were found to be the most frequent multiple-system congenital anomalies, with Down syndrome (Table A2) having the highest prevalence, accounting for 33.3% of all syndromic anomalies. This is in accordance with the literature, as approximately 0.4–0.9% of neonates are born with a chromosomal anomaly, with Down syndrome further accounting for 1/700–1/800 live births, making it the most common trisomy and autosomal anomaly [13].

Regarding maternal risk factors and characteristics, studies have revealed that chromosomal anomalies are greater in those aged above 35; however, non-chromosomal anomalies are greater in those aged less than 35. As most of the anomalies found in this study were non-chromosomal, this could explain the results in our study showing neonates of mothers aged 20–35 having the highest prevalence of CAs [14,15]. This is further supported by the 77% of the mothers of babies with Down syndrome in our study being ≥ 35 years old.

The occurrence of birth defects was found to be more common amongst the Bahraini nationality (67.2%) in comparison to non-Bahrainis (32.8%), which is concordant with Bahraini mothers representing the majority of the participants in the study. As consanguineous marriages are a common tradition in the Middle East, it doubles the risk of developing CAs and inherited conditions, causing the prevalence of CAs in that particular population to be 1.7–2.8%, higher than the average population [16,17]. However, further research is necessary to determine if an association is present.

Our study found a higher rate of cesarean section deliveries (58.2%) among the neonates with CAs. Cesarean sections are performed based on relevant obstetric indications depending on maternal and fetal factors. However, several studies, including one by Wataganara et al., suggested that cesarean delivery for infants with anomalies can reduce birth trauma and improve outcomes, particularly for extremely premature babies and those with major anomalies requiring immediate NICU admission [18]. In our study, 35% of the neonates were born prematurely, suggesting that the high cesarean section rate is justified by the severity of the CA and potential benefits for both the mother and the baby. Another potential explanation is the influence of religious and cultural factors surrounding pregnancy termination. Islamic religious and legal frameworks impose strict limitations on abortion, permitting the procedure within the first 120 days of gestation primarily in cases where the mother’s life or health is at risk or when severe fetal abnormalities are diagnosed [19]. Given that many CAs are not detected until 18 to 20 weeks of pregnancy, termination may not be a viable option. In these cases, early knowledge of the condition facilitates timely preparation and collaborative decision making between healthcare providers and patients, including the consideration of cesarean delivery as an alternative management strategy [20].

Mothers with spontaneous conception and multiparity had a higher prevalence of CAs. Despite our paper being consistent with several studies regarding multiparity, it does differ from the current literature with regard to the method of conception [21,22]. Studies have shown that children born after assisted reproductive techniques, namely in vitro fertilization pregnancies (IVF), had a 25–50% increased risk of developing a birth defect. This is due to several factors, including the advanced age of the infertile couple, underlying infertility conditions, medications used in the process of IVF, and procedures such as embryo freezing and intracytoplasmic sperm injection (ICSI). ICSI, in particular, may introduce sperm abnormalities and cause cellular damage to the oocyte, leading to genetic defects and chromosomal abnormalities. These factors combined elevate the likelihood of birth defects in IVF pregnancies [23,24,25]. Although our results did not show a higher prevalence of CAs in IVF pregnancies, this may be attributed to the fact that the majority of the pregnant women included in this study had spontaneous conceptions, leading to an underrepresentation of IVF pregnancies in our sample.

It is important to note that the development of certain anomalies, such as genetic-related conditions, occurs spontaneously. Each pregnancy carries its own independent risk, and the chance of a future child having the condition remains independent of whether a previous child was affected by the majority of conditions [26]. This is seen in Table 2 where 97.7% of the mothers did not have a previous offspring with an abnormality.

In accordance with the current literature, our findings show a statistically significant association between pre-gestational hypertension, diabetes status, and CA prevalence. Both have been well documented, with studies attributing the increased risk in hypertensive mothers to hypertensive medications and uteroplacental insufficiency [4,27,28,29,30]. Moreover, studies show that diabetic mothers have an 8–12% increased chance of birthing a baby with CAs. Despite GDM occurring later in pregnancy, it can be considered an indication of longer-term metabolic imbalances before pregnancy [27,28].

Our analysis of the characteristics of babies with anomalies revealed a higher prevalence in males. Although the mechanism underlying this sex disparity remains unclear, several theories have been proposed. A theory by Lubinsky et al. states that male fetuses are more susceptible to developing anomalies during organogenesis than females, who may be more vulnerable during the blastocyst stage [31]. This theory aligns with the findings from several studies reporting a higher total female mortality rate during pregnancy compared to males, with male fetuses being more likely to survive despite having an anomaly [32,33,34,35]. However, it is essential to consider that the factors influencing the fetus’s survival with CAs are not solely dependent on sex.

The majority of babies born with CAs in our study were term (65%) and of normal birth weight (62.6%). Several studies, such as those conducted in Saudi Arabia, Qatar, and Morocco, are consistent with our findings [6,8,36]. Regarding neonatal birth weight, although our results are consistent with other studies, they conflict with the current presumption that preterm babies are at greater risk of CAs. This encourages a reassessment of this factor, as suggested by Narapureddy et al. [6]. 

### 4.3. Strengths and Limitations 

While this study provides valuable and updated insights into the prevalence of CAs in Bahrain’s biggest hospital and is the first to explore potential risk factors, some limitations are important to consider. Firstly, although SMC serves as the primary referral center for most CA cases, it is plausible that less severe anomalies may not warrant referral, resulting in their underrepresentation in our data and leading to an underestimation of the true prevalence of CAs in the broader population. Additionally, our study may not capture cases managed in private hospitals, as these institutions typically only refer cases that require intervention. Secondly, as the data were collected from handwritten registries, the accuracy of our findings is ultimately limited by the quality of the original data collection. Thirdly, alongside the exclusion of stillbirths, the registry only captured CAs identified at birth, excluding those diagnosed later in life; as a result, the true prevalence of CAs in Bahrain during this study period might be underestimated. Fourthly, our analysis was limited by the absence of data on several important maternal risk factors not routinely recorded at the hospital, including BMI, smoking status, alcohol consumption, consanguinity, and nutrient deficiencies. Lastly, as this paper is a unicenter study, the results may not fully represent national rates. Our limitations highlight the importance of creating a nationwide database on congenital anomalies and associated risk factors for future research.

## 5. Conclusions

This study provides updated insights into the prevalence of CAs in Bahrain, reporting a prevalence of 1.1%. These results can be used to guide healthcare policy and resource allocation by highlighting the need for enhanced antenatal screening and improved neonatal support, particularly for mothers with known risk factors. Early antenatal detection of major congenital anomalies is crucial for providing proper counseling to parents, planning for fetal or neonatal intervention, ensuring delivery at the appropriate facility, and allowing future prevention. To optimize the management of CAs, it is essential to establish a specialized database that meticulously tracks and analyzes these conditions. This will enable in-depth studies on anomalies and support the provision of enhanced counseling services to effectively assist mothers and families. Future research should utilize nationally representative samples and investigate additional maternal risk factors, such as BMI, consanguinity, smoking, and alcohol consumption.

## Figures and Tables

**Table 1:** Association between maternal and perinatal factors and congenital anomalies.

Variable	Group	Congenital Anomaly					
		Yes		No		Total Number	*p*-Value
		Number	%	Number	%		
Nationality	Bahraini	236	1.2%	18,981	98.8%	19,217	0.042
	Non-Bahraini	115	1.0%	11,661	99.0%	11,776	
Method of Conception ^1^	Spontaneous	310	12.6%	2153	87.4%	2463	0.000
	Induced	27	3.9%	661	96.1%	688	
Method of Delivery	Normal Vaginal Delivery	137	0.7%	20,312	99.3%	20,449	0.000
	Assisted Vaginal Delivery	11	4.5%	234	95.5%	245	
	Lower Segment Cesarean Section	206	1.9%	10,715	98.1%	10,921	
Diabetes Mellitus Status ^1^	Pre-gestational Diabetes Mellitus	6	1.1%	532	98.9%	538	0.000
	Gestational Diabetes Mellitus	33	4.3%	735	95.7%	768	
	None	336	1.1%	30,109	98.9%	30,445	
Hypertension ^1^	Yes	11	2.9%	362	97.1%	373	0.001
	No	343	1.1%	30,267	98.9%	30,610	
Gender	Male	203	1.2%	16,238	98.8%	16,441	0.015
	Female	144	0.9%	15,023	99.1%	15,167	
Multiple Gestations	Yes	16	1.6%	1004	98.4%	1020	0.166
	No	338	1.1%	30,257	98.9%	30,595	
Gestational Age at Birth	Preterm	124	2.7%	4415	97.3%	4539	0.000
	Term/Post-Term	230	0.8%	26,846	99.2%	27,076	

^1^ missing data: method of conception (*n* = 14); diabetes mellitus status (*n* = 10); hypertension (*n* = 10).

**Table 2:** Characteristics of mothers whose babies had congenital anomalies (*n* = 351).

Variable	Group	Number	Percentage (%)
Maternal Age	<20 years	5	1.4%
	20–35	243	69.2%
	≥35 years	103	29.3%
Maternal Parity	Primipara	119	33.9%
	Multipara	210	59.8%
	Grandpara	22	6.3%
Previous Baby with Congenital Anomaly	Yes	8	2.3%
	No	346	97.7%

**Table 3:** Characteristics of babies with congenital anomalies (*n* = 354).

Variable	Group	Number	Percentage (%)
Neonatal Birth Weight	Extremely Low	5	1.4%
	Very Low	17	4.8%
	Low	100	28.3%
	Normal	221	62.6%
	High	10	2.8%
	Not Recorded	1	0.3%
Neonatal Outcome	Discharged	265	74.9%
	Perinatal Death	66	18.6%
	In-hospital Death	23	6.5%

## Data Availability

The original contributions presented in this study are included in the article. Further inquiries can be directed to the corresponding author. **Acknowledments:** This article is a revised and expanded version of a paper [37] entitled “Congenital Anomalies in Bahrain: Prevalence and Predictive Factors in Pregnant Women”, which was presented at the Royal College of Obstetricians and Gynaecologists World Congress, Oman, 15 October 2024.

## Appendix A

**Table A1:** Distribution of congenital anomalies involving a singular system (*n* = 212).

System	Congenital Anomaly	Number of Cases	Percentage (%)
Musculoskeletal	Talipes Equinovarus	20	9.4
	Polydactyly	18	8.5
	Multiple MSK Congenital Anomalies	3	1.4
	Unclassified MSK Congenital Anomaly	3	1.4
	Achondroplasia	2	0.9
	Congenital Amputation	2	0.9
	Syndactyly	1	0.5
	Osteogenesis Imperfecta	1	0.5
	Upper Limb Phocomelia	1	0.5
Cardiology	Multiple Cardiac Congenital Anomalies	19	9.0
	Transposition of the Great Vessels	4	1.9
	Hypoplastic Left Heart Syndrome	3	1.4
	Septal Defects	2	0.9
	Patent Ductus Arteriosus	2	0.9
	Cardiac Arrhythmias	2	0.9
	Unclassified Cardiac Congenital Anomaly	2	0.9
	Coarctation of the Aorta	1	0.5
	Tetralogy of Fallot	1	0.5
	Double Inlet Ventricle	1	0.5
	Tricuspid Regurgitation	1	0.5
	Ectopia Cordis	1	0.5
Oral and Maxillofacial	Ankyloglossia	10	4.7
	Cleft Lip and Cleft Palate	9	4.2
	Cleft Lip	8	3.8
	Cleft Palate	4	1.9
	Neonatal Teeth	2	0.9
	Congenital Ranula Cyst	1	0.5
	Multiple Oral and Maxillofacial Congenital Anomalies	1	0.5
Central Nervous System	Anencephaly	5	2.4
	Hydrocephalus	5	2.4
	Multiple CNS Congenital Anomalies	4	1.9
	Arnold–Chiari Malformation	4	1.9
	Encephalocele	3	1.4
	Spina Bifida	3	1.4
	Microcephaly	2	0.9
	Meningoencephalocele	2	0.9
	Macrocephaly	1	0.5
	Sacrococcygeal Teratoma	1	0.5
Gastrointestinal	Diaphragmatic Hernia	12	5.7
	Anorectal Malformation	7	3.3
	Omphalocele	6	2.8
	Esophageal Atresia	1	0.5
	Congenital Umbilical Hernia	1	0.5
	Multiple GI Congenital Anomalies	1	0.5
Genitourinary	Multiple GU Congenital Anomalies	4	1.9
	Congenital Hydronephrosis	2	0.9
	Congenital Adrenal Hyperplasia	2	0.9
	Unclassified GU Congenital Anomaly	2	0.9
	Congenital Posterior Urethral Valve	1	0.5
	Bilateral Renal Agenesis	1	0.5
	Polycystic Kidney	1	0.5
	Multicystic Dysplastic Kidney	1	0.5
	Ambiguous Genitalia	1	0.5
Ear, Nose and Throat	Abnormal Ear	3	1.4
	Cystic Hygroma	3	1.4
Skin	Epidermolysis Bullosa	2	0.9
	Ichthyosis	1	0.5
	Supernumerary Nipples	1	0.5
	Unclassified Skin Congenital Anomalies	1	0.5
Respiratory	Congenital Cystic Adenomatoid Lung Malformation	2	0.9
Ophthalmology	Microphthalmos with Retinal Detachment	1	0.5

**Table A2:** Distribution of Syndromes (*n* = 66).

System	Congenital Anomaly	Number of Cases	Percentage (%)
Syndromes	Down Syndrome (Trisomy 21)	22	33.3
	Potter Syndrome	11	16.7
	Unclassified Syndromes	10	15.2
	Patau Syndrome (Trisomy 13)	5	7.6
	Edwards’ Syndrome (Trisomy 18)	3	4.5
	OEIS Syndrome	2	3.0
	Dandy–Walker Syndrome	2	3.0
	Goldenhar Syndrome	2	3.0
	VACTERL Syndrome	2	3.0
	Meckel-Gruber Syndrome	1	1.5
	Beckwith Wiedemann Syndrome	1	1.5
	Pierre Robin Sequence	1	1.5
	Prune Belly Syndrome	1	1.5
	Waardenburg Syndrome	1	1.5
	Mermaid Syndrome	1	1.5
	Oculocutaneous Albinism	1	1.5
